# Autonomous ultrasound scanning robotic system based on human posture recognition and image servo control: an application for cardiac imaging

**DOI:** 10.3389/frobt.2024.1383732

**Published:** 2024-05-07

**Authors:** Xiuhong Tang, Hongbo Wang, Jingjing Luo, Jinlei Jiang, Fan Nian, Lizhe Qi, Lingfeng Sang, Zhongxue Gan

**Affiliations:** ^1^ Academy for Engineering and Technology, Fudan University, Shanghai, China; ^2^ Intelligent Robot Engineering Research Center of Ministry of Education, Shanghai, China; ^3^ Institute of Intelligent Medical Care Technology, Ningbo, China

**Keywords:** ultrasound scanning, robotic system, posture recognition, point cloud, path planning, image servo control

## Abstract

In traditional cardiac ultrasound diagnostics, the process of planning scanning paths and adjusting the ultrasound window relies solely on the experience and intuition of the physician, a method that not only affects the efficiency and quality of cardiac imaging but also increases the workload for physicians. To overcome these challenges, this study introduces a robotic system designed for autonomous cardiac ultrasound scanning, with the goal of advancing both the degree of automation and the quality of imaging in cardiac ultrasound examinations. The system achieves autonomous functionality through two key stages: initially, in the autonomous path planning stage, it utilizes a camera posture adjustment method based on the human body’s central region and its planar normal vectors to achieve automatic adjustment of the camera’s positioning angle; precise segmentation of the human body point cloud is accomplished through efficient point cloud processing techniques, and precise localization of the region of interest (ROI) based on keypoints of the human body. Furthermore, by applying isometric path slicing and B-spline curve fitting techniques, it independently plans the scanning path and the initial position of the probe. Subsequently, in the autonomous scanning stage, an innovative servo control strategy based on cardiac image edge correction is introduced to optimize the quality of the cardiac ultrasound window, integrating position compensation through admittance control to enhance the stability of autonomous cardiac ultrasound imaging, thereby obtaining a detailed view of the heart’s structure and function. A series of experimental validations on human and cardiac models have assessed the system’s effectiveness and precision in the correction of camera pose, planning of scanning paths, and control of cardiac ultrasound imaging quality, demonstrating its significant potential for clinical ultrasound scanning applications.

## 1 Introduction

The heart, as one of the most critical organs in the human body, performs the essential functions of pumping blood and supplying oxygen. However, heart disease remains one of the major global health challenges. In the diagnosis and monitoring of heart disease, echocardiography (commonly known as cardiac ultrasound) has become the most frequently used non-invasive cardiac imaging technique due to its portability, real-time capability, lack of radiation risk, and low cost. Nonetheless, the traditional cardiac ultrasound (US) scanning process faces several challenges, including the susceptibility of image quality to the probe’s position and operator technique, poor reproducibility of handheld operations, physical strain on operators due to prolonged working periods, and potential risks of radiation and infection. These challenges limit the efficiency and safety of echocardiography in clinical applications. With technological advancements, autonomous US scanning technology has shown immense potential for development. Integrating robotics, advanced image processing methods, and artificial intelligence can effectively reduce the operational burden on physicians and enhance the level of autonomy and intelligence in the diagnostic process. This evolution promises to address the current limitations of echocardiography, offering improvements in both clinical efficiency and patient safety.

Most autonomous US scanning robots execute the scanning process in two stages: initially, the robot analyzes pre-operative human body data to locate the US probe scanning area and path, and determines the probe’s initial scanning posture. Subsequently, the robot adjusts and optimizes the probe’s position and contact force in real-time based on interactive information with the human body during the scanning process (such as force-position data and USimages), thereby ensuring patient safety and achieving high-quality US imaging. This dual-phase approach leverages advanced robotics and real-time feedback mechanisms to enhance the precision and safety of US diagnostics.

The localization of scanning areas and the planning of scanning paths are fundamental to achieving autonomous US scanning. With the advancement of visual sensing technology, vision-based guidance techniques have gained widespread application in the field of medical robotics. For instance, Graumann et al. (2016) utilized an RGB-D camera to collect point cloud data of the patient’s surface, then manually selected regions of interest (ROI) for alignment with preoperative CT images to plan the US probe’s scanning trajectory. However, reliance on CT images and manual registration models increased the overall cost of US scanning. To simplify the process, Kojcev et researchers (2017) demonstrated the repeatability of vision-based robotic US acquisition by manually delineating scanning trajectories in RGB-D images. To further advance the autonomy of US robotic scanning and achieve autonomous localization of US scanning areas, Huang et al. (2018, 2019) and Lan and Huang (2018) implemented autonomous segmentation of scanning areas utilizing image color features and established the probe’s contact posture using the normal vectors of point clouds. Moreover, researchers including Mustafa et al. (2013) and Li et al. (2018) segmented the navel and nipples in RGB-D images based on skin color pixel differences to locate initial scanning positions. However, recognition methods based on color features are susceptible to interference from environmental colors and lighting conditions. In response to this challenge, image processing techniques driven by deep learning have shown significant advantages. For example, Tan et al. (2023) employed a keypoint detection method based on YOLO-Pose to identify and locate breast ROIs in RGB images. However, this method assumes that the body is aligned and symmetrical relative to the camera, and significant localization errors can occur if the body is tilted relative to the camera (Okuzaki et al., 2024). Researchers like Soemantoro et al. (2023) used the YOLOv5 object detection model to train on manually selected quadrilateral windows of scanning areas in color images, achieving automatic localization of ROIs without analyzing the accuracy of positioning. In addition, most of the above path planning methods intuitively select scanning paths in a 2D image and then map them to the 3D surface of the human body. However, this approach may lead to large differences in the distances between the actual paths (Huang et al., 2018; 2019; Lee et al., 2018; Welleweerd et al., 2020).

The control of scanning pose compensation and the optimization of the US window are crucial for maintaining good contact between the probe and the body surface, as well as stable window quality. During the autonomous US scanning process, the magnitude and variation of contact force significantly impact the quality and stability of US images, as observed by Ipsen et al. (2021). Researchers have proposed various control algorithms to maintain a constant contact force, including impedance control (Suligoj et al., 2021), admittance control (Tan et al., 2022; Wang Z. et al., 2023), and PID control (Ma et al., 2021; Zhang et al., 2021; 2022). Moreover, due to the significant fluctuations in window quality during cardiac US scanning, researchers have introduced several US image servo control algorithms to ensure stable, high-quality imaging. Chatelain et al. (2016, 2017) introduced an US confidence map integrated into a visual servo framework to enhance window quality and target tissue tracking through probe scanning direction adjustments. Jiang et al. (2020, 2022) used US confidence map technology to accurately estimate the optimal probe direction at contact points, thereby improving image quality at specific locations. However, the US confidence map serves only as a means to quantify US credibility, and the US window adjusted based on the confidence map may not represent the actual optimal image. Moreover, real-time calculation of the confidence map demands high computational performance from the host computer (Jiang et al., 2023). Despite significant advancements in autonomous US scanning control technology, the practicality of robotic systems is limited due to their reliance on complex environmental sensing and control algorithms (Li et al., 2021; von Haxthausen et al., 2021; Roshan et al., 2022).

Considering the current state of cardiac US autonomous scanning technology, which remains in its infancy with few existing studies on US robots possessing both autonomous path planning and scanning pose compensation capabilities (Ferraz et al., 2023), this paper addresses these technical challenges with two hypotheses: Firstly, ensuring the human body remains symmetrical and centered within the camera’s positioning window is crucial for the accuracy and stability of ROI localization. This objective can be achieved by utilizing camera-based recognition of human keypoints followed by pose correction. Secondly, maintaining symmetry in the areas of lower quality on both sides of the cardiac US window helps ensure that the probe’s window remains perpendicular to the heart throughout the autonomous scanning process. Given the limited precision of confidence map calculations, this study employs grayscale image analysis to assess the areas of lower quality within the cardiac US window for probe window correction. Furthermore, based on the aforementioned hypotheses and the practical needs of robotic operation, this research has developed a fully autonomous cardiac US scanning system aimed at advancing the diagnosis of heart diseases towards automation and intelligence. The primary contributions of this paper include:

i) To accurately segment human body surface point cloud data, an efficient processing method for complex raw point cloud data is introduced. Addressing the limitations of two-dimensional human posture localization in capturing complex spatial relationships and posture variations, an innovative camera posture adjustment method based on human posture recognition is proposed. The method ensures correct alignment of the viewing angle through camera pose correction based on human posture keypoints and their planar normal vectors. Additionally, a positioning strategy based on the geometric relationships of keypoints is presented for precise localization of the cardiac ROI.
ii) To ensure comprehensive, uniform, and smooth autonomous scanning of the probe over the body surface, a path planning method based on point cloud slicing and Non-Uniform Rational B-Splines (NURBS) curves is introduced. By equidistantly segmenting the projection of the body surface ROI point cloud on the camera coordinate system’s XOY plane and using NURBS curve techniques to fit the path within slices of discrete point clouds, the uniformity and smoothness of the scanning path are ensured, laying the foundation for high-quality cardiac US scanning.
iii) To address the issue of target displacement within the cardiac US window, a lightweight US image servo control algorithm has been developed. This algorithm, by analyzing the grayscale values of the cardiac edges, dynamically adjusts the angle of the cardiac center axis in real-time, ensuring the heart maintains its optimal position within the imaging window. Moreover, in response to the dynamically changing scanning environment, a scanning pose compensation method that integrates multiple sources of information has been proposed. By real-time integration of axial admittance control for position compensation and US image servo control for posture compensation on top of visually planned probe scanning pose information, the position and posture of the scanning probe are effectively optimized. This approach enhances the quality and stability of cardiac US imaging throughout the scanning process and offers a significant improvement in the efficiency and reliability of cardiac diagnostic procedures.

## 2 Materials and methods

### 2.1 System setup and design

#### 2.1.1 System setup

**FIGURE 1:**
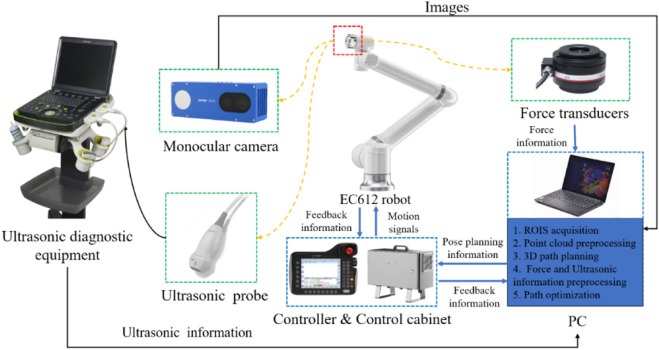
System setup.

As depicted in Figure 1, The cardiac US scanning robot consists of five major components as follows: a six-degree-of-freedom robotic arm (EC612, Elite Robotics, China), a US imaging system with a linear probe (Clover 60, Wison, China), a force/torque sensor (γ82, Decheng, China), an RGB-D camera (D132s, ChiSense, China), and a human heart model (BPH700-C, CAE, United States). The robot hardware system and functionality are shown in Figure 1, in which the RGB-D camera, the robot controller, the six-dimensional force sensors, and the PC communicate via an Ethernet switch. And the US image data is transferred from the device to the PC via an HDMI interface. The robot control and image processing algorithms are implemented on a PC (AMD Ryzen 7 5800H, 3.20 GHz, 16 GB RAM). The development environment is PyCharm, based on Open3d, geomdl, OpenCV2 libraries, and the device SDK for algorithm development.

#### 2.1.2 Definition of probe scan pose

**FIGURE 2:**
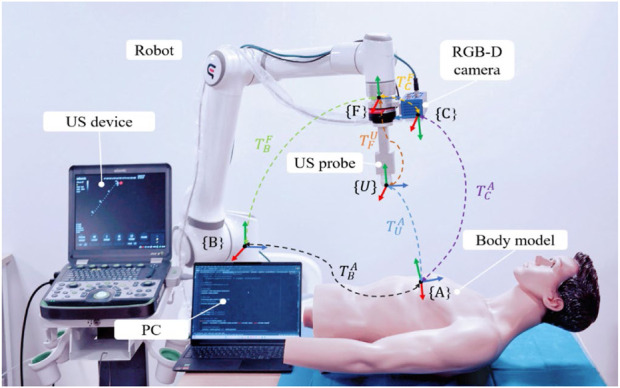
System coordinate transformation.

To standardize the spatial pose description, we unified the probe’s coordinate space and the human body surface’s ROI into the robot base coordinate system. As depicted in Figure 2, the robot base coordinate system is {B}, the robot end coordinate system is {F}, the camera coordinate system is {C}, the probe coordinate system is {U}, and the coordinate system at the scan target point P is {A}. Homogeneous transformation from the robot end effector coordinate system {F} to the robot base coordinate system {B} is derived through forward kinematics. Transformation from the camera coordinate system {C} to the robot end effector coordinate system {F} is achieved via hand-eye calibration using a calibration plate, as detailed by Marchand et al. (2005). The center point of the end of the US probe to the end of the robot $T_{F}^{U}$ can be obtained by tool calibration with a tool. Consequently, the transformation from the scan target point to the robot base coordinate system, $T_{B}^{A}$ , is delineated as follows:

$$T_{B}^{A}=T_{B}^{F}T_{F}^{C}T_{C}^{A}$$ (1)

And the left of Eq. 1 can be expressed as follows:

$$T_{B}^{A}=\left[\begin{array}{cc} R_{B}^{A} & P_{B}^{A}\\ 0 & 1 \end{array}\right]$$ (2)

where $P_{B}^{A}$ denotes the coordinate of the scan target point P(x,y,z), the rotation matrix $R_{B}^{A}$ can be converted to obtain the Euler angle about the ZYX axes:

$$\begin{array}{ll} rz & =\arctan2\left({R_{B}^{probe}}_{21},{R_{B}^{probe}}_{11}\right)\\ ry & =-\arcsin{R_{B}^{probe}}_{31}\\ rx & =\arctan2\left({R_{B}^{probe}}_{32},{R_{B}^{probe}}_{33}\right) \end{array}$$ (3)

Consequently, according to Eqs 2, 3, the initial attitude of the probe at the target point can be expressed as:

$$P_{probe}=\left(x,y,z,rx,ry,rz\right)$$ (4)

#### 2.1.3 Workflow

The robotic scan workflow can be divided into the following three primary phases:

(i) Information-gathering

This initial phase involves calibrating the camera, probe, and robotic system, as well as gathering the necessary data before US scanning to ensure that all equipment is ready and accurately aligned.

(ii) Planning of scanning paths and probe poses

First, with the help of human posture recognition technology, the camera is adjusted to be directly above the human posture recognition plane. Then, the human body surface point cloud is preprocessed, and the ROI point cloud is segmented autonomously based on the keypoints of the human body. Next, the scanning path is planned by projecting slices of the point cloud combined with NURBS curve fitting. Finally, the scanning attitude of the US probe is determined by passing the normal vector of the path points and the path direction.

(iii) Compensatory control of probe scanning poses

In the autonomous scanning stage, the robot real-time fuses the axial contact force-based guide position compensation and the US image servo-control-based attitude compensation in the vision-guided position information, realizing the real-time optimization of the scanning position and US view window.

**FIGURE 3:**
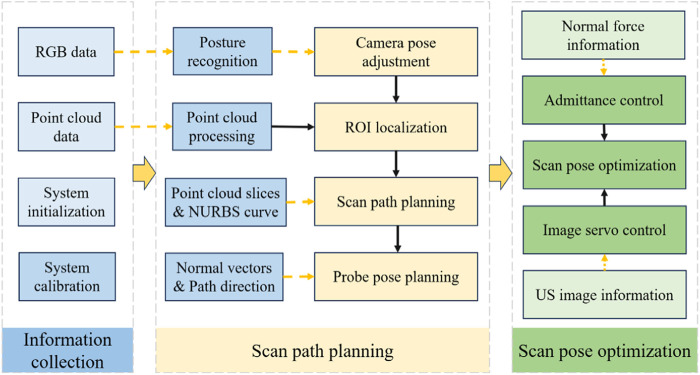
Workflow of the autonomous US scanning process.

The entire workflow is depicted in Figure 3, which illustrates the synergy of the three phases mentioned above.

### 2.2 ROI localization based on human posture recognition

**FIGURE 4:**
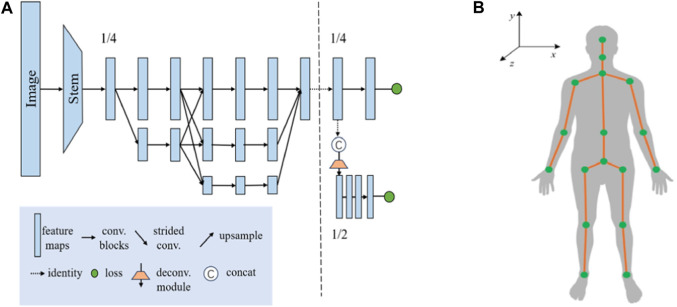
Human pose recognition algorithm: **(A)** HigherHRNet network structure model, and **(B)** Human critical points of COCO dataset A. Adjustment of the camera’s positioning pose.

In the field of human posture recognition, although algorithms such as YOLO-Pose and YOLOv5 have demonstrated rapid and efficient characteristics in target detection, they exhibit certain limitations in accurately recognizing complex human postures and localizing keypoints. This study selects HigherHRNet as the keypoint recognition algorithm, primarily due to its high accuracy and robustness in handling multi-scale human postures and precise keypoint localization. These capabilities are achieved through the use of high-resolution feature pyramids and multi-resolution fusion techniques. The structure of the HigherHRNet network model is depicted in Figure 4. Moreover, the advantages of HigherHRNet have been validated on the COCO dataset, currently recognized as the standard testing platform for complex posture recognition. The performance of HigherHRNet on the COCO dataset, especially in cases of partial occlusions, demonstrates its exceptional capability in processing a variety of complex postures (Cheng et al., 2020). This provides a reliable scientific basis for its application in ROI localization within cardiac US scanning.

#### 2.2.1 Adjustment of the camera’s positioning pose

To address the issue of inaccurate ROI localization caused by the body’s inclination relative to the camera, this study proposes a camera pose adjustment technique based on a human posture recognition algorithm. This method precisely corrects the camera’s pose by analyzing keypoints of human posture and their planar normal vectors, achieving consistent alignment of the camera’s viewpoint. This alignment ensures the body remains symmetrical within the camera’s positioning window, effectively overcoming the limitations of two-dimensional image localization techniques in capturing complex spatial relationships and changes in posture. The specific steps include:

i) Key point positioning

**FIGURE 5:**
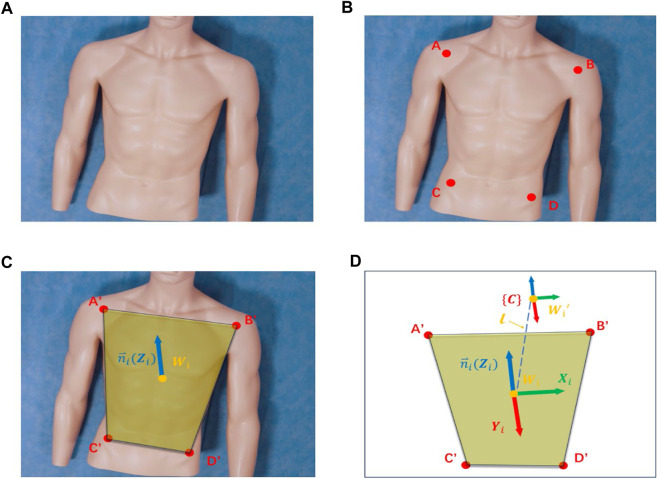
Camera pose adjustment: **(A)** Input RGB image. **(B)** Key point identification. **(C)** Schematic diagram of the human body posture plane. **(D)** Spatial diagram of camera adjustments.

Utilize a human keypoint detection model trained on HigherHRNet to precisely identify four main keypoints of the human body in color images: left shoulder A, right shoulder B, left hip C, and right hip D, as shown in Figure 5B. These points’ cloud positions in three-dimensional space are calculated, providing critical coordinate information for subsequent plane fitting.

ii) Plane fitting

Employing the least squares method, a plane representing the human posture is fitted based on the coordinates of the four keypoints, calculating the normal vector ${\vec{n}}_{i}$ of the plane. Further, the projections of these keypoints on the human posture plane are denoted as $A^{\prime },B^{\prime }{,C}^{\prime }\;{\text{an}d\;D}^{\prime }$ , with $W_{i}$ as the geometric center of these projection points' coordinates, as depicted in Figure 5C.

iii) Camera pose calculation

The origin of the camera coordinate system is set at position ${W_{i}}^{\prime }$ , directly above the point $W_{i}$ at a distance of l mm. The Z-axis of the camera coordinate system is directed along ${\vec{n}}_{i}$ and passes through the point $W_{i}$ , the X-axis direction is aligned with $\vec{A^{\prime }B^{\prime }}$ , and the Y-axis direction is calculated through the cross-product, as illustrated in Figure 5D.

iv) Camera Pose Adjustment

Following the guidance from steps 1 to 3, the robotic arm executes iterative adjustments of the camera’s pose. During this process, by comparing the pose difference with a predefined threshold, it is determined whether the condition for stopping adjustment has been met, thereby achieving precise correction of the camera’s pose.

#### 2.2.2 Point cloud segmentation and registration

i) Background filtering

**FIGURE 6:**
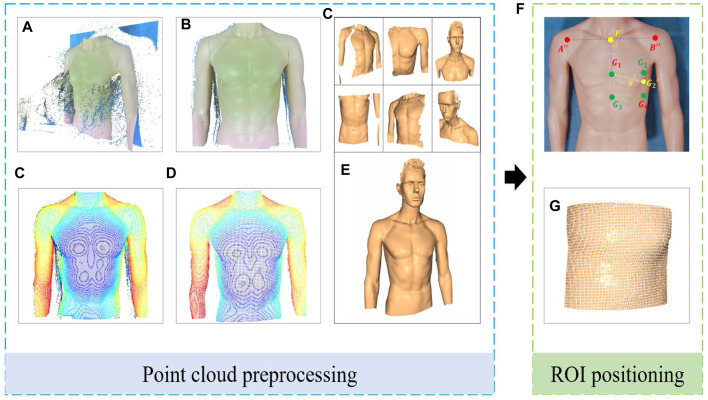
Point cloud processing and ROI localization steps: **(A)** Raw point cloud data. **(B)** Background filtered point cloud. **(C)** Point cloud after downsampling. **(D)** Denoised point clouds. **(E)** Multi-point cloud registration. **(F)** Schematic diagram of ROI localization. **(G)** Segmented ROI point cloud.

As shown in Figure 6A, the raw point cloud data of the human body captured by the RGB-D camera is voluminous and contains significant noise. Given that the position of the operating table and the robotic arm is relatively fixed, this paper utilizes a pass-through filter on the Z-axis dimension of the world coordinate system to segment and filter the point cloud data. This process retains only the point cloud related to the human body, as depicted in Figure 6B.

ii) Point cloud downsampling

Although background filtering can reduce the number of point clouds to some extent, the remaining data volume is still substantial, impacting the system’s ability to process point clouds efficiently. To decrease data density while preserving the integrity of the point cloud’s geometric structure, voxel filtering is used for data downsampling, as depicted in Figure 6C.

iii) Point cloud denoising

To enhance the positioning accuracy of path points within the ROI, it is necessary to further denoise the reconstructed point cloud model. This study employs a combined approach of statistical filtering and DBSCAN clustering to remove outliers. The DBSCAN clustering method is used to specify density criteria to identify clusters, removing small clusters and scattered points. This is further combined with statistical filtering to eliminate outliers significantly different from neighboring points, thereby smoothing the point cloud surface while preserving data structure, as depicted in Figure 6D. This dual-filtering approach demonstrates higher robustness when processing noise data of varying densities and shapes in human bodies.

iv) Multi-point cloud registration

To capture the complete surface morphology of the human body and supplement partial information missed from a single viewpoint, this study proposes a method of multi-viewpoint cloud capture and registration. This method involves collecting point cloud data from multiple viewpoints for 3D reconstruction and using Fast Point Feature Histograms (FPFH) features and the Random Sample Consensus (RANSAC) algorithm for global registration. Subsequently, the Iterative Closest Point (ICP) algorithm is employed for fine registration to achieve high-precision matching of point cloud data, as depicted in Figure 6E. The optimized mathematical model of the ICP algorithm can be expressed as follows:

$$T_{i+1}=\arg\min_{T}\sum_{j}{\left\|q_{i}-T\left(p_{i}\right)\right\|}_{2}^{2}$$ (5)

In this context, $T_{i+1}$ represents the transformation matrix obtained after the i+1 iteration, $T\left(p_{j}\right)$ denotes the mapped position of point $p_{j}$ in the source point cloud under the current transformation, and $q_{j}$ is the corresponding point in the target point cloud to $p_{j}$ .

#### 2.2.3 ROI localization and segmentation

Based on an in-depth analysis of the ROI in cardiac US and the clinical experience of US physicians, this study identifies a clear geometric positional relationship between the surface ROI area and human keypoints. Drawing on existing research findings (Hao et al., 2024), we propose a novel ROI localization method that relies on acquired RGB images and point cloud information to precisely locate human keypoints, thereby accurately determining the ROI position using the coordinate relationships of these keypoints. The specific method is as follows:

i) ROI localization

Initially, within the camera coordinate system, the projection points of the two shoulder keypoints on the XOY plane, $A^{\prime \prime }$ , $B^{\prime \prime }$ , are determined. Based on these two points, the four boundary points of the ROI, $G_{1}$ , $G_{2}$ , $G_{3}$ and $G_{4}$ , are defined, as depicted in Figure 6F. Here, $G_{1}$ and $G_{4}$ serve as the starting and ending points of the scanning path, respectively. The perpendicular bisector of the line segment $A^{\prime \prime }B^{\prime \prime }$ , $G_{1}E$ is used as the left-right dividing line of the body, while the Euclidean distance between $A^{\prime \prime }$ and $B^{\prime \prime }$ , $l_{A^{\prime \prime }B^{\prime \prime }}$ , serves as a basis for determining the overall size of the ROI range. Furthermore, it is set that $G_{1}G_{2}$ are parallel to $G_{3}G_{4}$ , and $G_{1}G_{3}$ and $G_{2}G_{4}$ are parallel to EF. On this basis, the length of $G_{1}E$ is set to 0.4 $l_{A^{\prime \prime }B^{\prime \prime }}$ , the length of $G_{1}G_{3}$ is set to 0.25 $l_{A^{\prime \prime }B^{\prime \prime }}$ , the angle formed between the line segment $G_{1}G_{2}$ and $A^{\prime \prime }B^{\prime \prime }$ is θ, and the distance between $G_{1}G_{3}$ and $G_{2}G_{4}$ is 0.3 $l_{A^{\prime \prime }B^{\prime \prime }}$ .

ii) ROI point cloud segmentation

After defining the boundary points of the ROI, we extract all the human body point clouds projected onto the XOY plane and located within the four boundary points $G_{1}$ , $G_{2}$ , $G_{3}$ , and $G_{4}$ to complete the point cloud segmentation of the cardiac US ROI. As depicted in Figure 6G, this shows the point cloud of the cardiac ROI when *θ* is 0.

### 2.3 Planning of scanning paths and probe poses

#### 2.3.1 Planning of scanning paths

**FIGURE 7:**
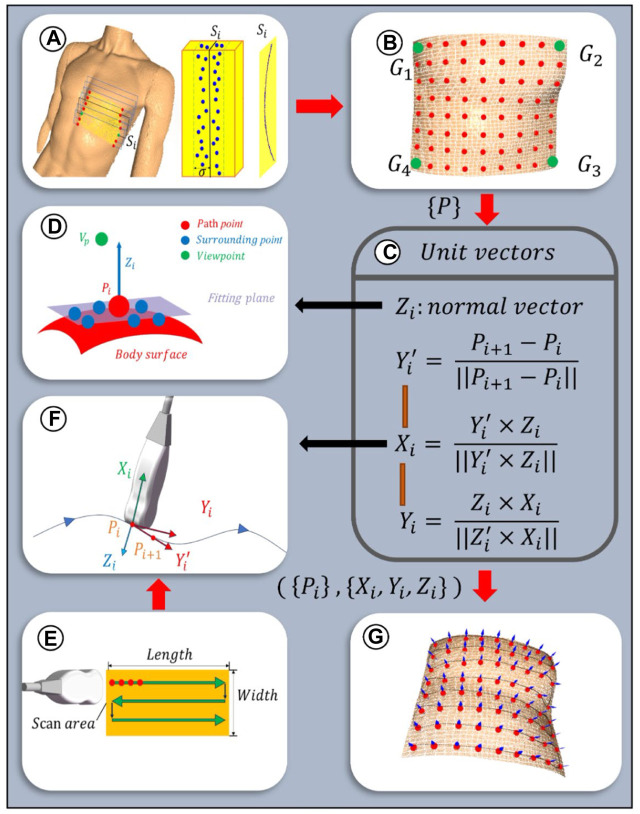
Scanning paths and probe poses planning algorithm: **(A)** Path planes within ROI. Discrete point cloud within the ith slice with a width of $\frac{\sigma }{2}$ mm. Path fitting in the ith slice $S_{i}$ . **(B)** Isometric selection of path points. **(C)** Formulation of probe orientation at path points. **(D)** Schematic diagram of normal vector calculation. **(F)** Schematic diagram of probe orientation calculation. **(E)** Schematic diagram of 2D path planning. **(G)** Scanning paths and probe poses are planned.

To ensure the paths uniformly cover the cardiac ROI space, the path planes are selected equidistantly based on the XOY plane. Given the sparse distribution of point clouds on the designated path plane, path slicing is utilized to acquire nearest neighbor points to populate the points required for path fitting. The distance between adjacent paths is related to the width of the US (US) probe, denoted as d mm, with the probe’s scanning direction oriented along the longer dimension of the scanning area. To increase the overlap between paths, an additional overlap measure c is defined. As depicted in Figure 7A, path planes are evenly positioned along the shorter edge of the ROI projection, adhering to a predetermined path spacing of d-c mm. Subsequently, path slices required for path fitting are created by extracting points on both sides of these path planes at a distance of $\frac{\sigma }{2}$ mm.

Given the discrete nature of the point clouds within the path slices, a third-order Non-Uniform Rational B-Splines (NURBS) curve is employed to fit the path curves, thereby more effectively adapting to changes in the surface curvature of the body (Zhang et al., 2023). This is achieved by adjusting weights and selecting appropriate control points to enhance consistency with the characteristics of the point cloud data. The fitting equation for the third-order NURBS curve is as follows:

$$P\left(t\right)=\frac{\sum_{i=0}^{n}N_{I,3}\left(t\right)\cdot w_{i}\cdot P_{i}}{\sum_{i=0}^{n}N_{I,3}\left(t\right)\cdot w_{i}}$$ (6)

$$E={\;\sum_{i=1}^{n}\;\left\|P|\left(t_{i}\right)|-|P_{i}\right\|}^{2}$$ (7)

$$N_{i,3}\left(t\right)=\frac{1}{6}\left[{\left(1-t\right)}^{3},3t^{3}-6t^{2}+4,-3t^{3}+3t^{2}+3t+1,t^{3}\right]$$ (8)

where $P\left(t\right)$ is the point on the curve; $N_{i,3}\left(t\right)$ represents the third-order NURBS basis function, E is the control vertex optimization objective function, and $P_{i}$ is the coordinates of the control vertices; $w_{i}$ is the weight associated with each control vertex; n is the number of control vertices; t is the curve parameter, ranges with [0,1].

As depicted in Figure 7E, the scanning paths are designed along a two-dimensional plane, with the baseline from the ROI boundary $G_{1}$ to $G_{3}$ . To ensure the continuity of the scanning process, the probe automatically moves to the starting point of the next path after completing one path and scans in the opposite direction. This process continues uninterrupted until all predetermined paths have been thoroughly scanned. As depicted in Figure 7B, extract appropriate path points along each scanning path.

#### 2.3.2 Planning of initial probe scanning poses

To optimize the US probe’s angle of incidence to be nearly perpendicular, thereby enhancing image clarity (Wang Y. et al., 2023), the probe’s initial pose was established based on the normal vector to the skin surface at path points. Given the local geometric characteristics of the point cloud data, a KD tree structure was constructed for efficient indexing of neighboring point sets for each path point. Centroid alignment is applied to these point sets to eliminate offset, followed by linear fitting using the least squares method to derive local plane equations. From these equations, normal vectors were extracted and further refined through optimization based on the residual sum of squares, ensuring a precise approximation of local surface features. The equation for this specific optimization process is delineated as follows:

$$\min_{c,n,\left|\left|n\right|\right|=1}\sum_{i=1}^{m}{\left({\left(P_{i}-C\right)}^{T}Z_{i}\right)}^{2}$$ (9)

where C is the centroid of a set of neighboring points and $\left(P_{i}-C\right)$ denotes a decentered point.

The unit normal vectors obtained from Eq. 9 are bidirectional, and direction unity is achieved by the path point to viewpoint vector coinciding with the normal vector (Figure 7D), which is satisfied:

$$Z_{i}\cdot \left(v_{p}-P_{i}\right)>0$$ (10)

In order to ascertain the axial angle rz of the probe, its broadside is aligned such that it remains parallel to the scanning path’s tangent. As depicted in Figure 7F, the tangent vector $Y_{i}^{\prime }$ determined from the sum of two adjacent points $P_{i+1}$ and $P_{i}$ , may not align perpendicularly with the normal vector. The unit positive direction vector for the X and Y coordinate axes at the target point’s location is determined through cross-product operations as illustrated in Figure 7C. Subsequently, the rotation transformation matrix for the probe’s coordinate system to the robot arm’s base coordinate system, $R_{B}^{probe}=\left(X,Y,Z\right)$ , is formulated by arranging them in the order of $X_{i}$ 、 $Y_{i}\text{ and }Z_{i}$ . This rotation matrix is then translated into Euler angles rz,ry,rz following Eq. 3, facilitating the acquisition of the probe’s initial pose information at the target point, denoted as $P_{probe}=\left(x,y,z,rx,ry,rz\right)$ . Figure 7G presents the planning diagram for all scanning paths and probe poses within the ROI.

### 2.4 Compensatory control of probe scanning poses

#### 2.4.1 Axial position compensation based on admittance control

To ensure effective US penetration through the skin and the acquisition of high-quality cardiac images, this study builds upon previous research on force-controlled US scanning by applying an admittance control algorithm for precise axial position compensation of the probe (Jiang et al., 2024). This admittance control guides the robotic arm to adjust the probe’s position along the Z-axis based on the changes in axial contact force between the probe and the skin, thereby enhancing the system’s adaptability to complex environments and the stability of cardiac US imaging. The expression for admittance control is as follows:

$$M_{d}\left({\ddot{Z}}_{d}-\ddot{Z}\right)+B_{d}\left({\dot{Z}}_{d}-\dot{Z}\right)+K_{d}\left(Z_{d}-Z\right)=F_{c}$$ (11)

$${∆z=Z}_{d}-Z$$ (12)

where $M_{d},B_{d},$ and $K_{d}$ are the inertia, damping, and stiffness parameters of impedance control, respectively; $F_{c}$ is the difference between the actual force and the reference force; Z is the initial positioning position in the axial direction; $Z_{d}$ is the desired position in the axial direction; ∆z is the compensated axial position.

Thus, according to Eq. 4 and Eq. 12, the pose after position compensation of the probe can be expressed as follows:

$$P_{probe}^{\prime }=\left(x,y,z+∆z,rx,ry,rz\right)$$ (13)

#### 2.4.2 Orientation compensation based on servo control of cardiac US image edges

**FIGURE 8:**
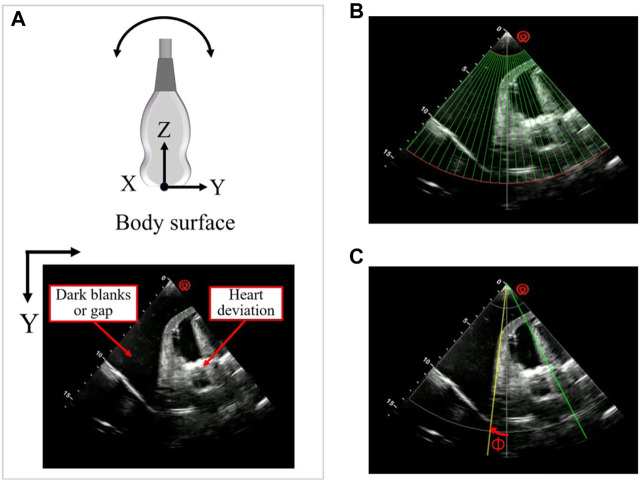
Schematic diagram of US image servo control **(A)** Artifacts and cardiac deviation in the echocardiography window. **(B)** The observation area of the US window is divided into 24 sub-regions. **(C)** Schematic diagram of cardiac US image servo control.

As depicted in Figure 8A, although the probe scanning posture, employing position compensation technology based on visual positioning and admittance control, can achieve clear imaging of the heart, artifacts and cardiac displacement may still occur within the US window. In order to optimize the cardiac US window in real-time during the scanning process, this study proposes an US image servo-control based attitude compensation strategy. This strategy aims to ensure the accuracy and quality of cardiac US imaging by adjusting the probe attitude to reduce or eliminate artifacts and offsets during imaging.

In the US sectoral window, the upper layer typically comprises the skin fat layer, whereas the lower layer often exhibits lower image quality. The observation area is delineated as an annulus within the polar coordinate system, defined by Ω = [ $r_{\min}$ , $r_{\max}$ ]×[ $\theta_{\min}$ , $\theta_{\max}$ ], situated between these two layers. To enhance the precision of window optimization, this area is partitioned into *m* sub-areas, where the central angle of each subregion is α , as depicted in Figure 8B. A threshold, σ, is established based on the mean grayscale value across the observation area to distinguish the valid region (values above σ) from the invalid region (values below σ). The mean grayscale value, $\mu_{j}$ , of each sub-region, along with the desired steering angle, Ф, for compensation, are computed as follows:

$$\mu_{j}=\frac{1}{N}\int_{\theta_{i}}^{\theta_{i+1}}\int_{r_{\min}}^{r_{\max}}I\left(r,\theta \right)rdrd\theta ,i=1,2,\cdot \cdot \cdot ,m$$ (14)

$$Ф=\frac{K\cdot \left(a-b\right)\cdot \alpha }{2}$$ (15)

where $I\left(r,\theta \right)$ is the grayscale value of the pixel at the polar position $\left(r,\theta \right)$ , *N* is the number of pixels in each sub-region, *K* is the control gain, a、b represent the amount of the invalid region adjacent to the center region on both sides, respectively, obtained by comparing the grayscale values calculated according to Eq. 14 with the threshold value σ, and α is the angle of the center of the circle of the sub-region.

Finally, based on Eq. 13 and Eq. 15, the pose of the probe after attitude compensation, considering probe position compensation, can be expressed as follows:

$$P_{probe}^{\prime \prime }=\left(x,y,z+∆z,rx+Ф,ry,rz\right)$$ (16)

As depicted in Figure 8C, the robot arm adjusts the probe, causing it to rotate around its X-axis coordinate system to the desired steering angle Ф, thereby aligning the probe’s gray centerline to the desired yellow centerline. Throughout this procedure, Ф (targeting an expected value of 0°) serves as the control parameter. Orientation compensation is executed in real time via an iterative method, designed to enhance image quality.

The overall US image servo control algorithm flow can be seen in the pseudo-code in Table 1:

**TABLE 1:** Cardiac US image servo control algorithm flow.

**Algorithm** 1: Cardiac US image servo control (ISC)
**Input**: observation window parameters **Ω** (center coordinates *p*, inner radius $r_{\min}$ , outer radius $r_{\max}$ , start angle $\theta_{start}$ , end angle $\theta_{end}$ ), sub-regions_amount *m*
**Output**: steering angle Ф
1: masks, α ← divide_observation_regionTomasks( *p,* $r_{\min},r_{\max},$ ; $\theta_{start}$ *,* $\theta_{end}$ *, m*)
α = $\frac{\theta_{end}-\theta_{start}}{m}$ ;//the central angle of each subregion
2: **While** readVideoFrames() = True **do**
3: grayscale_frame ← convert_frameTograyscale()
4: grayscale_values $\mu_{j}$ , mean $\mu_{0}$ ← mask_extract_grayscalevalues(grayscale_frame, masks)
$\mu_{j}=\frac{1}{N}\int_{\theta_{i}}^{\theta_{i+1}}\int_{r_{\min}}^{r_{\max}}I\left(r,\theta \right)rdrd\theta ,i=1,2,\cdot \cdot \cdot ,m;$ //The grayscale value of the subregion
5: **if** $\mu_{0}$ > 0.16 **then**
6: *a, b* ← calculate_invalid_subregions( $\mu_{j}$ , $\mu_{0}$ )
//The amount of the invalid zone adjacent to the center region on both sides
7: calculate_steering_angle (*a, b,* α *, K*)
Ф = $\frac{K\cdot \left(a-b\right)\cdot \alpha }{2}$ ;//Steering angle
8: **end if**
9: scan_control(∆z, Ф); //∆z is axial position compensation
10: **end while**

#### 2.4.3 Establishment of a coordination mechanism

Given that the tasks of ISC and admittance control operate independently under separate threads, maintaining the contact force within a consistent range is prioritized. At the initial point, the robotic system employs admittance control to determine the compensated position for the entire path in the normal direction. Throughout the scanning process, it persistently assesses the contact force’s magnitude and the real-time variations in image quality. Should the image quality fall below the desired standard, the US window is adjusted to improve image quality without compromising the stability of the contact force. Conversely, if the contact force exceeds a predetermined threshold, the robotic system prioritizes the adjustment of the Z-axis position to maintain a safety level of contact force.

## 3 Experimentation and results analysis

**FIGURE 9:**
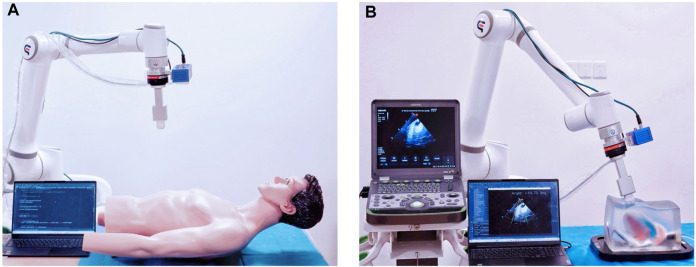
Experimental test platform: **(A)** experimental platform based on visual planning. **(B)** experimental platform for autonomous scanning control.

To evaluate the methods and algorithms proposed in this study, experiments were conducted using the cardiac scanning robot system depicted in Figure 1. The experimental setup includes the robot’s control frequency set at 30 Hz, force information read at a frequency of 200 Hz, and cardiac US image processing at a frequency of 50 Hz. As shown in Figure 9, the experiments were divided into three groups across two models, aiming to assess the precision of camera adjustment localization, the quality of path fitting, and the imaging outcomes of US servo control.

### 3.1 Evaluation of localization accuracy under camera pose adjustment

To verify the effectiveness of the camera pose adjustment method based on human posture recognition algorithms proposed in this study, and to evaluate its positioning accuracy, experiments were conducted using a human model as the recognition target. Camera positioning adjustments were tested, as depicted in Figure 9A. The experiment was set with an iteration count of 5, a predetermined camera pose difference threshold of 10 mm, and a camera distance setting height of 670 mm. The adjustment experiment was conducted under 20 different initial camera poses. Following the operational steps described in Section 2.2 1, the camera was adjusted to the optimal positioning pose, and the pose of the camera, as well as the coordinate information of the four identified human keypoints (A, B, C, and D) were recorded.

**FIGURE 10:**
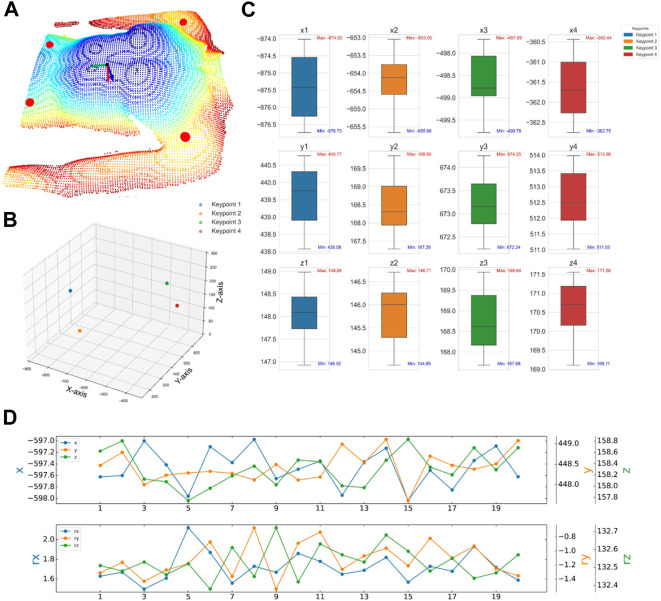
Camera pose adjustment experiment **(A)** Normal vectors of human point clouds, keypoints, and human pose planes after a set of camera adjustment experiments. **(B)** Spatial location of four human critical points after 20 sets of experiments. **(C)** Camera poses after 20 sets of experiments. **(D)** Four keypoints of the human body after 20 sets of experiments.

As depicted in Figure 10A presents the point cloud of the human body after the camera was adjusted to the pose of the first set of experiments, the distribution of the point cloud of the four keypoints, and the direction arrows of the camera pose. Figure 10B presents the distribution of the coordinate positions of the four keypoints in 3D space for the 20 sets of experiments. To present the experimental results more intuitively, Figure 10C presents the 3D coordinate box plots of the four keypoints detected after camera adjustment, and the maximum Euclidean deviations of the four keypoints were calculated from the data to be 2.36 mm, 2.91 mm, 1.60 mm, and 2.19 mm, respectively. Meanwhile, Figure 10D summarizes the corresponding camera-adjusted pose parameters, including the x, y, and z coordinates and the rx and ry rotation angles, which are −596.73 ± 1.13 mm, 447.99 ± 1.12 mm, 157.72 ± 0.72 mm, and 188 ± 0.38°, −1,16 ± 0.41°,132.06 ± 0.84°, respectively. These experimental results demonstrate the high precision and repeatability of camera pose adjustments under various initial viewpoints. Since ROI localization is based on the positioning of keypoints and human posture within the camera’s view, these results also indirectly prove the effectiveness of the camera adjustment method based on human posture recognition in solving two-dimensional ROI localization issues.

### 3.2 Evaluation of path-fitting performance

**FIGURE 11:**
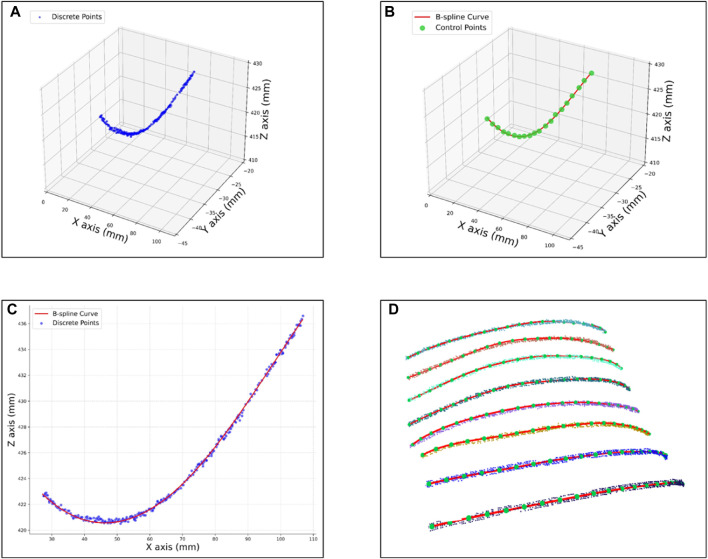
Scanning path fitting experiments **(A)** Discrete points of a slice. **(B)** Curve fitting after selecting control vertices within a slice. **(C)** Projections of fitted curves and discrete point clouds onto the XOZ plane. **(D)** Path fitting of discrete point clouds within all slices.

Ensuring the uniformity and smoothness of scanning paths within the cardiac Based on an in-depth analysis of the ROI in cardiac US and the clinical experience of US physicians, this study identifies a clear geometric positional relationship between the surface ROI area and human body keypoints. during the autonomous cardiac US scanning process is crucial for achieving stable and fluid scanning movements. For this purpose, this study provides a detailed demonstration of path fitting within the cardiac ROI and validates the rationality of the path design through quality assessment. The initial steps involve projecting the point cloud data within the ROI onto the XOY plane and setting the angle between the path and the projected line segment of shoulder keypoints *θ* to zero. Within the projected boundary of the ROI, path slices 0.8 mm wide are selected at equal intervals, and point cloud data extracted from these slices are used for path fitting. Figure 11A shows the discrete point cloud set corresponding to the first path slice in three-dimensional space.

Using the Open3D and geomdl libraries, these point cloud path slices were fitted with third-order Non-Uniform Rational B-Splines (NURBS) curves. To accurately capture the path contours, 20 control points were selected for each path, and the weights of all control points were uniformly set to 1, with the results shown in Figure 11B. For a visual demonstration of the fitting effect, Figure 11C displays the projection of the discrete point cloud and its fitting curve for the first path slice on the XOZ plane. Finally, Figure 11D comprehensively presents the point cloud data, control points, and fitting curves for eight path slices, fully showcasing the fitting results.

**FIGURE 12:**
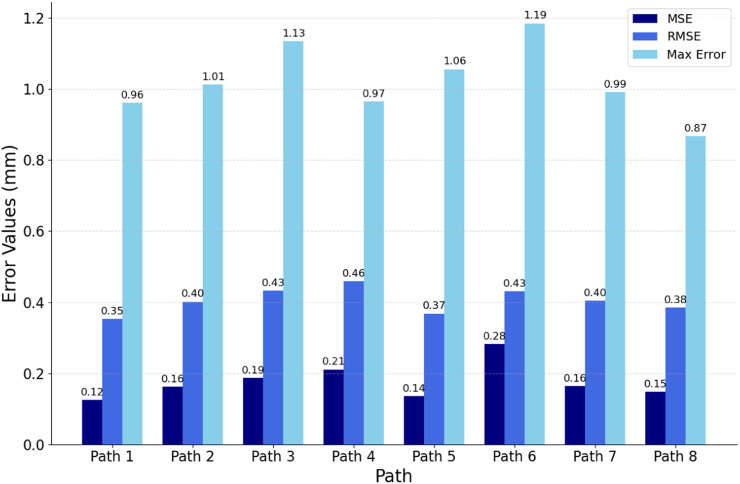
Evaluation of fitted path curves.

To comprehensively evaluate the fitting quality within the path slices, this experiment quantified the bias by computing the Euclidean spatial distances from the discrete point clouds within the path slices to their nearest curve smoothing points. As depicted in Figure 12, the histograms of mean square error (MSE), root mean square error (RMSE), and maximum error (MA) between the discrete point clouds and the fitted curves within the selected eight path slices are displayed. The results show that the MSE is ≤0.28 mm, the RMSE is ≤0.46 mm and the MA is ≤1.19 mm. These metrics indicate that the overall path deviation is in full compliance with the planning requirements of the autonomous scan paths, thus confirming the effectiveness of this path-fitting method in path quality control.

### 3.3 Evaluation of cardiac US imaging quality

During the autonomous cardiac US scanning process, relying solely on visual positioning and force perception control proves insufficient for ensuring the quality of imaging at the window center. As demonstrated in Figure 9B, this study conducted automatic scanning tests on a cardiac model to evaluate the effectiveness of the Image Steering Control (ISC) algorithm in reducing cardiac imaging displacement within the US window. In the experimental setup, the US gain was fixed at 80, with probe position and posture information updated at a frequency of 30 Hz to facilitate real-time autonomous control. The experiments were conducted both with the ISC mode enabled and disabled, where the robotic arm controlled the probe to perform continuous autonomous scanning along eight predefined paths within the heart’s ROI. By comparing the changes in grayscale values of the US images and the ideal steering angles within the observation area under these two modes, this experiment accurately assessed the effectiveness of the ISC technology in enhancing the quality of US imaging and its precision in adjustment.

**FIGURE 13:**
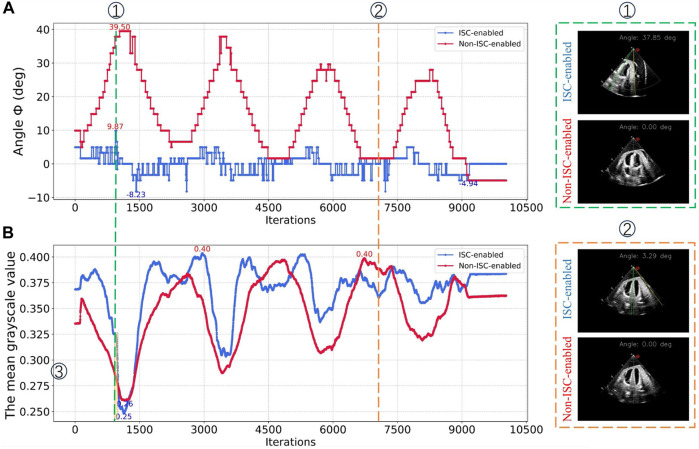
Image quality assessment experiment **(A)** In ISC-enabled mode versus non-ISC-enabled mode, the probe scans record the desired steering angle. **(B)** In both modes, the probe scans record the mean grayscale value within the viewing window.

As depicted in Figure 13A records the variation of the desired steering angle during probe scanning in both modes, while Figure 13B shows the variation of the mean grayscale value of the US image during probe scanning. The peaks and valleys of these fluctuations correspond to the locations of the two ends of the heart on the trajectory, respectively. It was observed that scanning the probe to the right resulted in a significant increase in the desired steering angle when ISC was not enabled, while it decreased when scanning to the left. In addition, regardless of whether ISC was enabled or not, the mean grayscale value of the US images decreased significantly when the probe was scanned to the right, while it increased when the probe was scanned to the left. In particular, when the desired steering angle was close to 0°, this indicated that the heart was in the center of the viewing window when imaging was optimal.

As can be seen from the gray value curves in Figure 13B, the mean grayscale value of the images obtained during ISC-enabled scanning is generally higher than that observed during non-ISC-enabled scanning, especially in the regions outside the path transitions. In addition, the fluctuation of the desired steering angle is smoother when ISC is enabled, indicating higher and more stable image quality. The yellow transparent area in Figure 13B reveals a phenomenon: when the compensated steering angle is too large, the mean grayscale value of ISC enabled may drop sharply. At the orange marker line, the probe pose based on visual navigation is close to ideal, showing that the image quality remains good with or without ISC enabled. However, at the green marker line on the left side of Figure 13, where the probe is further away from the heart, the image quality without ISC enabled and with ISC enabled shows a significant difference, with a mean grayscale value difference and desired steering angle difference of 0.041 and 39.5°, respectively. The experiments showed that the application of the ISC algorithm during the cardiac US scan can effectively perform orientation compensation and significantly improve the image quality in the US view window.

## 4 Discussion

Currently, autonomous US scanning robots typically face challenges such as insufficient precision in localizing the ROI, difficulty in ensuring window quality, high operational complexity, and adaptability issues in dynamic scenes. For autonomous cardiac US scanning, this study introduces camera pose adjustment based on human posture recognition, enabling the system to precisely locate the cardiac ROI based on human keypoints in various scenarios. Compared to methods based on color feature extraction (Lan and Huang, 2018; Huang et al., 2019) and positioning solely on unadjusted two-dimensional images (Soemantoro et al., 2023; Tan et al., 2023; Okuzaki et al., 2024), this approach offers stronger anti-interference capabilities, providing a more accurate and stable imaging benchmark. By projecting three-dimensional point cloud data and processing it through equidistant slicing, the system in this study effectively handles irregular point cloud data in three-dimensional space and generates uniform, smooth scanning paths through NURBS curve fitting. The innovation of this method lies in providing a simple and precise path-planning solution for complex body surface geometries, significantly enhancing the regularity and adaptability of scanning paths compared to path selection based solely on RGB images (Huang et al., 2018; 2019; Lee et al., 2018; Welleweerd et al., 2020). The servo control strategy proposed in this study, based on admittance control and cardiac image edge correction, allows the system to monitor and adjust the US imaging window in real-time to optimize image quality. This strategy effectively optimizes the pose during scanning, improving the quality and stability of cardiac imaging. Compared to complex calculations for optimizing probe pose based on confidence maps (Jiang et al., 2022; 2023), this strategy reduces the system’s adaptation cost to dynamic changes, achieving real-time optimization of the US window and offering new solutions for enhancing US imaging quality.

Although this study has made significant progress in the development of a cardiac US scanning robot system, there are still some limitations. To reduce design and operational costs, the scanning process primarily focused on axial position compensation and optimization of imaging quality within the window, while strategies for avoiding obstacles within the window (such as ribs) need further exploration. Future work will consider using out-of-plane angle adjustments to effectively avoid obstacles and explore integrating multi-dimensional freedom control strategies for the probe, to enhance the system’s adaptability to dynamic environments. Additionally, the current manual application of US coupling gel increases the risk of infection. Future work will develop an automatic gel application device to enhance the system’s convenience and autonomy.

## 5 Conclusion

In this study, we have successfully developed an autonomous cardiac US scanning robot system capable of independently identifying the cardiac scanning area, planning scanning paths, and probe positions before the scan, and optimizing the pose in real-time during the scanning to acquire high-quality cardiac US images. Utilizing camera adjustment technology based on human posture recognition, the system can correct the camera’s positioning pose. After precise extraction of the human point cloud through point cloud processing and registration techniques, the system achieves accurate localization and segmentation of the Region of Interest (ROI) based on human keypoints. Subsequently, using point cloud slicing and Non-Uniform Rational B-Splines (NURBS) curve fitting techniques, it obtains uniform and smooth scanning paths. Furthermore, a servo control method for cardiac image edge correction was proposed to optimize the cardiac US window. During the scanning process, the system integrates an innovative servo control strategy based on admittance control and cardiac image edge correction, enhancing the quality and stability of autonomous cardiac US scanning imaging. Through rigorous experimental validation, our research not only demonstrates the effectiveness and precision of the system and its key technologies but also highlights its significant potential for clinical applications.

## Data Availability

The original contributions presented in the study are included in the article/Supplementary material, further inquiries can be directed to the corresponding authors.
